# The Spring Festival Is Associated With Increased Mortality Risk in China: A Study Based on 285 Chinese Locations

**DOI:** 10.3389/fmed.2022.761060

**Published:** 2022-03-02

**Authors:** Guanhao He, Min Cai, Ruilin Meng, Jianxiong Hu, Ke Peng, Zhulin Hou, Chunliang Zhou, Xiaojun Xu, Yize Xiao, Min Yu, Biao Huang, Lifeng Lin, Tao Liu, Jianpeng Xiao, Weiwei Gong, Ruying Hu, Junhua Li, Donghui Jin, Mingfang Qin, Qinglong Zhao, Yiqing Xu, Weilin Zeng, Xing Li, Cunrui Huang, Lei Si, Xingfen Yang, Wenjun Ma

**Affiliations:** ^1^Guangdong Provincial Institute of Public Health, Guangdong Provincial Center for Disease Control and Prevention, Guangzhou, China; ^2^School of Public Health, Southern Medical University, Guangzhou, China; ^3^Guangdong Provincial Center for Disease Control and Prevention, Guangzhou, China; ^4^National Clinical Research Center for Cardiovascular Diseases, Fuwai Hospital Chinese Academy of Medical Sciences, Shenzhen, China; ^5^Jilin Provincial Center for Disease Control and Prevention, Changchun, China; ^6^Hunan Provincial Center for Disease Control and Prevention, Changsha, China; ^7^Yunnan Provincial Center for Disease Control and Prevention, Kunming, China; ^8^Zhejiang Provincial Center for Disease Control and Prevention, Hangzhou, China; ^9^Department of Public Health and Preventive Medicine, School of Medicine, Jinan University, Guangzhou, China; ^10^School of Public Health, Sun Yat-sen University, Guangzhou, China; ^11^Faculty of Medicine, The George Institute for Global Health, University of New South Wales, Sydney, NSW, Australia

**Keywords:** Spring Festival, mortality risk, China, vulnerable population, attributable fraction (AF)

## Abstract

**Background:**

The Spring Festival is one of the most important traditional festivals in China. This study aimed to estimate the mortality risk attributable to the Spring Festival.

**Methods:**

Between 2013 and 2017, daily meteorological, air pollution, and mortality data were collected from 285 locations in China. The Spring Festival was divided into three periods: pre-Spring Festival (16 days before Lunar New Year's Eve), mid-Spring Festival (16 days from Lunar New Year's Eve to Lantern Festival), and post-Spring Festival (16 days after Lantern Festival). The mortality risk attributed to the Spring Festival in each location was first evaluated using a distributed lag nonlinear model (DLNM), and then it was pooled using a meta-analysis model.

**Results:**

We observed a dip/rise mortality pattern during the Spring Festival. Pre-Spring Festival was significantly associated with decreased mortality risk (ER: −1.58%, 95%CI: −3.09% to −0.05%), and mid-Spring Festival was unrelated to mortality risks, while post-Spring Festival was significantly associated with increased mortality risk (ER: 3.63%, 95%CI: 2.15–5.12%). Overall, a 48-day Spring Festival period was associated with a 2.11% (95%CI: 0.91–3.33%) increased mortality. We also found that the elderly aged over 64 years old, women, people with cardiovascular disease (CVD), and people living in urban areas were more vulnerable to the Spring Festival.

**Conclusion:**

Our study found that the Spring Festival significantly increased the mortality risk in China. These findings suggest that it is necessary to develop clinical and public health policies to alleviate the mortality burden associated with the Spring Festival.

## Introduction

Several studies have reported that mortality dips before a symbolically meaningful occasion such as Christmas, the mid-autumn Festival, or Jewish holiday of Passover and peaks just afterward (1). For instance, Phillips et al. showed that Jewish mortality fell sharply below the expected level just before Passover and rose by an equal amount above the expected immediately afterward (2). They also found that the mortality among Chinese in the USA dips by 35.1% in the week before the mid-autumn Festival and peaks by the same amount (34.6%) in the following week (3).

However, some other studies showed that symbolically meaningful occasions such as holiday or birthday not only just postponed death but also significantly increased the mortality risk (4). Phillips et al. reported the occurrence of 4.65% more cardiac and 4.99% more noncardiac deaths than expected during the Christmas/New Year's holidays (5). Knight et al. also demonstrated the “Christmas Holiday Effect” on cardiac mortality in the Southern Hemisphere with seasonality opposite to that in previous reports (6). The possible reasons behind the holiday effect may be lifestyle changes, emotional stress associated with holidays, increased particulate pollution, an inappropriate delay in seeking medical care, etc.

The Spring Festival, known as Lunar New Year, is the most important holiday for Chinese around the world. The celebration commonly lasts for 15 days, starting on the evening before the 1st day of the first lunar month and ending at the Lantern Festival on the 15th day. Similar to western festival like Christmas, in this festival, people would have a large number of parties with family and friends. Owing to the festival celebrations, most Chinese would abruptly change their regular lifestyles into indulged life patterns, such as overeating, excessive drinking, irregular sleep, and overexcitation. Moreover, air pollution would be deteriorated in the Spring Festival due to the increased coal burning and fireworks (7). Third, some patients might take longer time to receive medical help or initiatively delay medical treatment until after the festival. Those behavioral, psychological, and environmental changes may affect morbidity and mortality.

However, a very few studies investigated this important topic. Recently, Lin et al. reported that hospital mortality risk in patients admitted to internal medicine departments during the Chinese Spring Festival was the highest in Taiwan region, and they conjectured that scarce medical resources and decreased quality of care in the hospital during the Spring Festival holidays may contribute to increased mortality (8). Nonetheless, no research has yet examined the mortality pattern of general population during the Spring Festival. Therefore, there are many questions to be answered on this topic: Is there a death dip/rise pattern during the Spring Festival? Does the Spring Festival effect on mortality really exist rather than just postponement with a dip/rise mortality pattern? Are the effects modified by individual or regional characteristics?

To gain new insights into the abovementioned gaps, we collected a large data set from 258 Chinese counties/districts in mainland China to explore the mortality pattern during the Spring Festival, quantify the mortality effect of the Spring Festival, and disentangle the Spring Festival effect from temperature and air pollution. Our study will be helpful for policy making to prevent excess mortality risks during the Spring Festival in China.

## Materials and Methods

### Study Location

**Figure 1:**
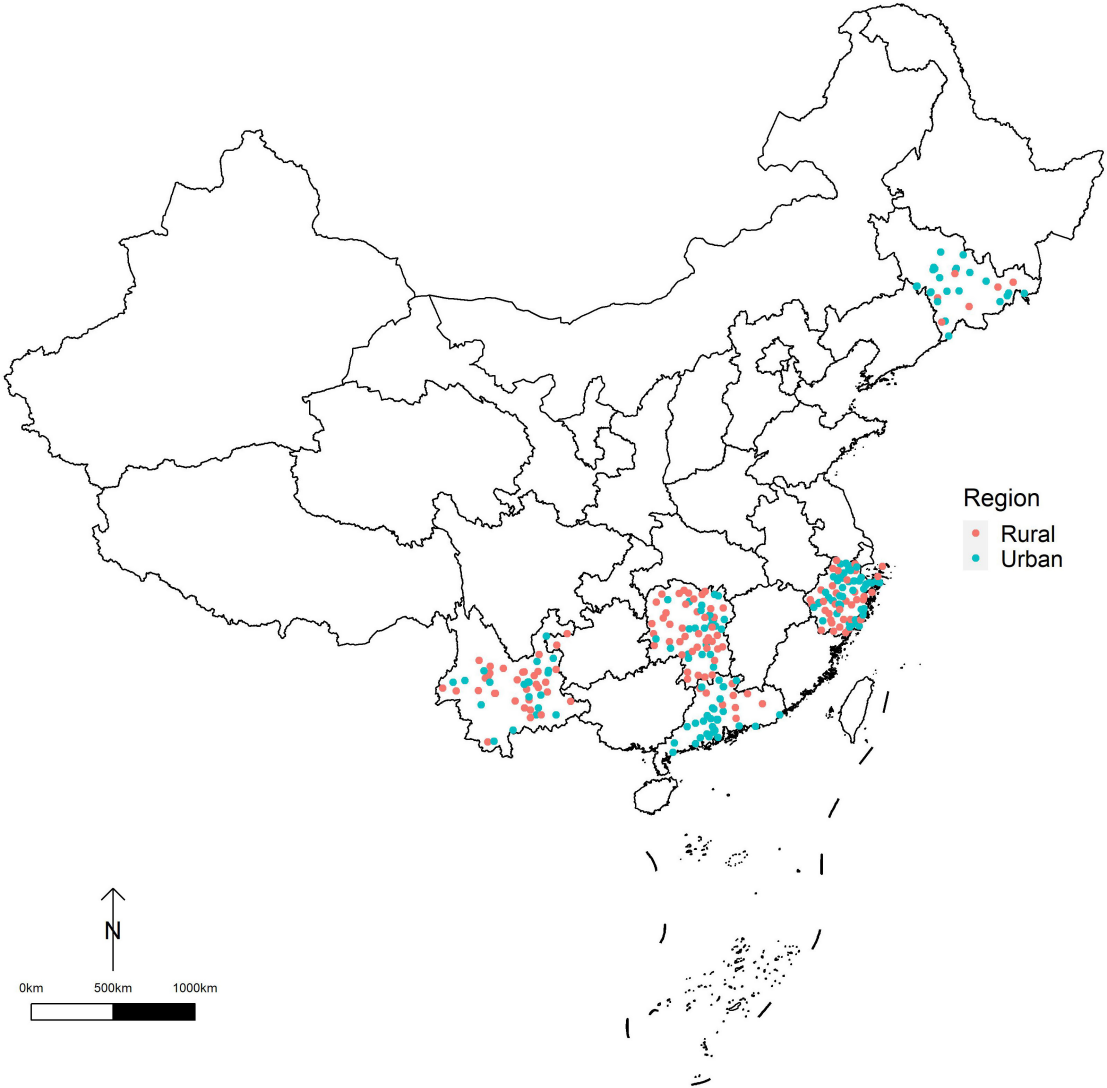
The geographical distribution of 285 study locations in China.

This study was based on a database of 285 locations (counties/districts) in Yunnan, Guangdong, Hunan, Zhejiang, and Jilin provinces in China. To ensure adequate statistical power, only the locations with population sizes over 200,000 or annual mortality rates >4‰ were selected in this study (9). We classified the 285 locations into two regions: urban districts (125 locations) and rural counties (160 locations) (Figure 1).

### Data Collection

Daily mortality data in each study location from Yunnan, Guangdong, Hunan, Zhejiang, and Jilin provinces (from January 1, 2013 to December 31, 2017) were collected from the corresponding provincial Centers for Disease Control. Daily mortality data included the date of death, gender, age, and the cause of death. All deaths were classified into groups based on primary diagnosis coded by International Classification of Diseases, 10th Revision (ICD-10) including total non-accidental causes (codes: A00-R99), cardiovascular disease (CVD, codes I00-I99), cerebrovascular disease (CED, codes I60-I69), and respiratory disease (RESP, codes J00-J98). Daily nonaccidental mortality data were further stratified by gender and age (0–64 or ≥65 years).

Meteorological data included daily mean temperature (°C) and relative humidity (RH, %). Meteorological data from 698 climate stations across China were derived from the China Meteorological Data Sharing Service System (http://data.cma.cn/). The Australian National University Splines (ANUSPLIN), an interpolation package based on the thin plate smoothing spline function, was employed to interpolate daily mean temperature and RH at 0·01° × 0·01° resolution across China (10). Their prediction accuracies have been verified (9, 11). Daily meteorological data of all 285 locations were extracted from the interpolated daily corresponding grid.

PM_10_ was selected as a representative of air quality during the study period, and it was adjusted as a potential confounder. Daily average PM_10_ was obtained from the National Urban Air Quality Real-time Publishing Platform (http://106.37.208.233:20035/), which is administrated by the China National Environmental Monitoring Center. As some study locations were not covered by the air quality monitoring system, we employed a land use regression model to estimate daily PM_10_ at each location, in which daily mean temperature, daily RH, latitude, longitude, altitude, population density, road density, types of land use, and the gross domestic product per capita were used as predictors. This method could achieve a good of fitness with 73.90% of R^2^, and root mean square error (RMSE) was 16.49 μg/m^3^.

### Statistical Analysis

#### The Definition of the Different Phases of the Spring Festival Period

Similar with Knight (6), the mid-Spring Festival was defined as the period from Lunar New Year's Eve to Lantern Festival (the 15th day of the first lunar month). To observe the dip/peak mortality pattern around the mid-Spring Festival, 16 days before and after the mid-Spring Festival were also included in an analysis and defined as pre-Spring Festival (16 days before Lunar New Year's Eve) and post-Spring Festival (16 days after Lantern Festival). To exclude the impact of death postponement until Symbolically Meaningful Occasions, we also estimated the total effect of pre-Spring, mid-Spring, and post-Spring Festivals. To minimize the impact of mortality seasonality, 16 days before pre-Spring Festival, and 16 days after post-Spring Festival (a total of 32 days) rather than a whole year were defined as control period.

#### Descriptive Analysis

In descriptive analyses, average mortality was calculated for each phase of the Spring Festival period. Mortality was reported as mean deaths with SDs. We stratified daily mortality by the cause of death (CVD, CED, and RESP), sex (male and female), age (0–64 years, 65 years over), and region (urban and rural). Scatter diagrams were used to describe the mean numbers of deaths, temperature, RH, and PM_10_ for each lunar calendar date.

#### Analyzing the Effects of the Spring Festival on Mortality

A two-stage analytic approach was used for an analysis. In the first stage, we applied a distributed lag nonlinear model (DLNM) linked with a quasi-Poisson distribution function to estimate the associations of the Spring Festival period with the mortality in each location. Several confounders, such as temperature, PM_10_, seasonal and long-term trends (a natural cubic B spline function of time with 2 degrees of freedom (df) per year), RH (a natural cubic B spline function with 3 df), and days of a week, were controlled. In the second stage, we combined the location-specific association using a meta-analysis, and the national relative risk (RR) of the Spring Festival was obtained. Then, the excess relative risk (ERR) was calculated by RR – 1, which was used to represent the mortality risks of the Spring Festival in this study. The best linear unbiased prediction (BLUP) of the location-specific cumulative associations between the Spring Festival and death was obtained using the previously fitted meta-analytical model. The rationale of the BLUP approach was a trade-off between the location-specific association and the pooled association, which can improve the preciseness of prediction, especially in locations with a small number of deaths.

To identify the possible vulnerable subpopulation, we conducted a stratification analysis by age (<65 years, ≥65 years), gender and diseases (CVD, CED, and RESP), and region (rural and urban).

Similar to the method proposed by Gasparrini (12), we further estimated the attributable fraction (AF) and attributable number (AN) of mortalities of the different phases of the Spring Festival in each location as follows:


(1) $$\begin{array}{l} AF=1-\exp\left(-\beta \right) \end{array}$$

(2) $$\begin{array}{l} AN=n\times AF \end{array}$$

where β refers to the regression coefficient of the Spring Festival and *n* refers to the total number of deaths in each location. Then, we summed the number of deaths attributable to the Spring Festival in all locations. The total AF was gained by dividing the total number of attributable deaths with the total number of deaths.

In addition, the Spring Festival-related excess death in China per year was estimated as follows (13):


(3) $$\begin{array}{l} \text{Excess death }=\text{ Pop }\times \text{ Daily Death Rate }\times \text{ AF }\times 48\text{ days } \end{array}$$

*Pop* referred to as the population of China in 2015 (2015 is in the central of our study period). Daily death rate was the daily nonaccidental cause mortality rate in 2015 and was calculated as the annual non-accidental cause mortality rate divided by 365. Pop and annual nonaccidental cause mortality rates were obtained from the Chinese Health Statistic Yearbook (14). The 48 days were referred to as the total Spring Festival in this study.

#### Sensitivity Analysis

A sensitivity analysis was conducted by changing degrees of freedom of time trend, a link function in DLNM, lag days of temperature or the Spring Festival, and the duration of the Spring Festival.

R software version 4.0.2 was used to perform data analysis. We applied “dlnm” package to construct the DLNM model and “mvmeta” package to conduct a multivariate meta-analysis.

## Results

**Figure 2:**
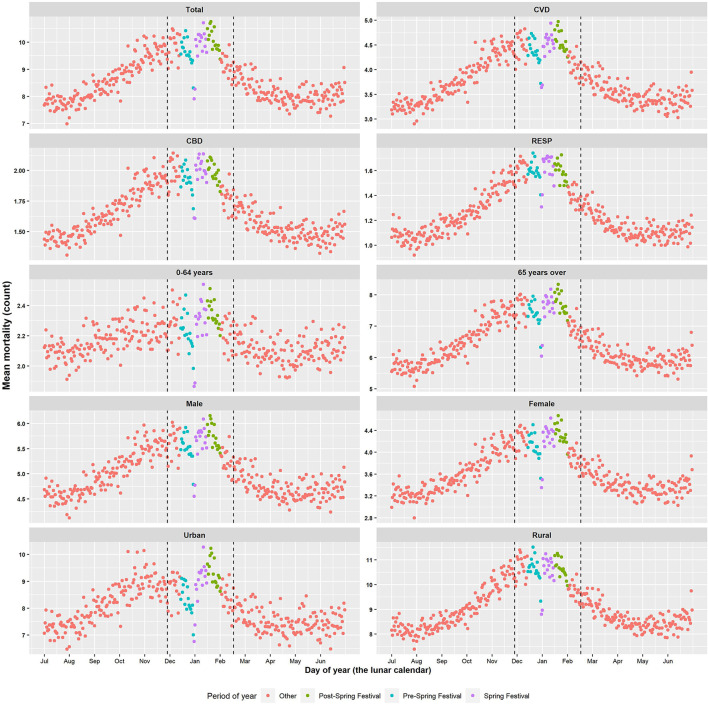
Daily mean mortality distribution during 2013–2017 by sex, age, cause of death, and region.

Table 1 and Figure 2 described the characteristics of mortality and environmental factors during the different phases of the Spring Festival. Generally, the average daily death counts in the total Spring Festival (9.84 ± 6.95) were higher than those in control period (9.49 ± 6.72). For the Spring Festival, the average daily counts in post-Spring Festival (10.08 ± 7.10) were higher than those both in the Spring Festival (9.81 ± 6.82) and pre-Spring Festival (9.63 ± 6.81). We also observed similar patterns for subgroups. Environmental factors such as average temperature, RH, and PM_10_ concentration were higher in control period than in the Spring Festival (Supplementary Figure S1).

**Table 1:** The characteristics of mortality and environmental factors during the different parts of the Spring Festival period.

	**The spring festival period**				**Control period^e^**
	**Pre-spring festival^a^**	**Mid-spring festival^b^**	**Post-spring festival^c^**	**Total spring festival^d^**	
**Total death**	9.63 (6.81)	9.81 (6.82)	10.08 (7.10)	9.84 (6.95)	9.49 (6.72)
**Age (year)**					
0-	2.22 (1.98)	2.27 (2.01)	2.34 (2.05)	2.28 (2.01)	2.24 (2.02)
65-	7.40 (5.61)	7.53 (5.70)	7.74 (5.84)	7.56 (5.72)	7.25 (5.50)
**Gender**					
Male	5.52 (4.16)	5.60 (4.22)	5.75 (4.31)	5.62 (4.23)	5.43 (4.11)
Female	4.11 (3.33)	4.21 (3.41)	4.3 (3 (3.50)	4.22 (3.42)	4.06 (3.29)
**Death of cause**					
CVD	4.37 (3.71)	4.45 (3.73)	4.58 (3.92)	4.47 (3.79)	4.28 (3.64)
CED	1.93 (1.95)	1.97 (1.96)	1.99 (1.97)	1.96 (1.96)	1.88 (1.88)
RESP	1.60 (1.84)	1.61 (1.85)	1.59 (1.83)	1.60 (1.84)	1.50 (1.76)
**Region**					
Urban	10.64 (7.09)	10.51 (7.09)	10.66 (6.98)	10.60 (7.06)	10.22 (6.89)
Rural	8.33 (6.19)	8.91 (6.60)	9.34 (7.18)	8.86 (6.68)	8.55 (6.37)
**Environmental factors**					
Temperature (°C)	5.68 (8.45)	7.32 (8.26)	10.09 (7.69)	7.70 (8.34)	8.95 (8.23)
Rh (%)	70.92 (13.20)	72.44 (14.58)	71.59 (14.81)	71.65 (14.23)	73.02 (13.74)
PM_10_	94.52 (60.29)	73.01 (45.45)	82.27 (41.93)	83.27 (50.64)	87.93 (49.46)

a *Mid-Spring Festival: from Lunar New Year's Eve to Lantern Festival (the 15th day of the first lunar month)*.

b *Pre-Spring Festival: 16 days before the mid-Spring Festival*.

c *Post-Spring Festival: 16 days after the mid-Spring Festival*.

d *Total Spring Festival: mid-Spring Festival + pre-Spring Festival + post-Spring Festival*.

e *Control Period: 16 days before pre-Spring Festival and 16 days after post-Spring Festival*.

Table 2 presented ERR in the different phases of the Spring Festival in China compared with control period. We found a death dip/rise pattern during the Spring Festival period, which meant that the mortality risk decreased during pre-Spring Festival (ERR: −1.58%, 95%CI: −3.09% to −0.05%) and increased during post-Spring Festival (ERR: 3.63%, 95%CI:2.15–5.12%). Totally, the Spring Festival period was associated with 2.11% (95%CI: 0.91–3.33%) increased mortality risk. For a subgroup analysis, the similar dip/rise pattern was also observed with much more fluctuations in rural locations than urban locations. However, we did not observe the total effect of the Spring Festival on the mortality risk in rural areas, populations aged 0–64 years old, and populations with CED or RESP. In addition, the total effects of the Spring Festival on mortality were higher in the elderly aged over 64 years old, women, CVD, and urban areas than in younger population aged 0–64 years old, CED, RESP, and rural areas.

**Table 2:** Excess relative risk (ERR, %) during the different phases of the Spring Festival period in China compared with control period.

	**Excess relative risk (%) and 95% CI**			
	**Pre-spring festival^a^**	**Mid-spring festival^b^**	**Post-spring festival^c^**	**Total spring festival^d^**
**Total**	−1.58 (−3.09, −0.05)^*^	−0.4 (−2.01, 1.23)	3.63 (2.15, 5.12)^*^	2.11 (0.91, 3.33)^*^
**Age (year)**				
0-	−2.46 (−4.79, −0.08)^*^	−1.26 (−4.13, 1.69)	2.86 (0.42, 5.37)^*^	1.27 (−0.72, 3.30)
65-	−1.35 (−3.03, 0.36)	−0.24 (−2.09, 1.65)	4.01 (2.41, 5.63)^*^	2.56 (1.23, 3.90)^*^
**Gender**				
Male	−1.82 (−3.49, −0.11)^*^	−1.48 (−3.36, 0.45)	3.00 (1.36, 4.67)^*^	1.97 (0.62, 3.34)^*^
Female	−1.36 (−3.23, 0.56)	0.65 (−1.59, 2.94)	4.76 (2.88, 6.68)^*^	2.68 (1.16, 4.22)^*^
**Death of cause**				
CVD	−1.37 (−3.32, 0.62)	−0.15 (−2.51, 2.27)	3.99 (2.05, 5.97)^*^	2.45 (0.85, 4.08)^*^
CED	−1.70 (−4.34, 1.01)	−0.02 (−3.11, 3.16)	3.74 (1.38, 6.15)^*^	1.81 (−0.37, 4.03)
RESP	−0.25 (−3.17, 2.75)	−1.29 (−4.71, 2.25)	1.88 (−0.69, 4.50)	2.14 (−0.14, 4.48)
**Region**				
Urban	0.99 (−0.63, 2.64)	−0.90 (−2.92, 1.16)	2.62 (0.87, 4.40)^*^	3.12 (1.61, 4.66)^*^
Rural	−4.75 (−7.40, −2.03)^*^	0.32 (−2.30, 3.02)	4.82 (2.29, 7.40)^*^	0.52 (−1.43, 2.50)

a *Mid-Spring Festival: from Lunar New Year's Eve to Lantern Festival (the 15th day of the first lunar month)*.

b *Pre-Spring Festival: 16 days before the mid-Spring Festival*.

c *Post-Spring Festival: 16 days after the mid-Spring Festival*.

d *Total Spring Festival: mid-Spring Festival + pre-Spring Festival + post-Spring Festival*.

* *p < 0.05*.

**Figure 3:**
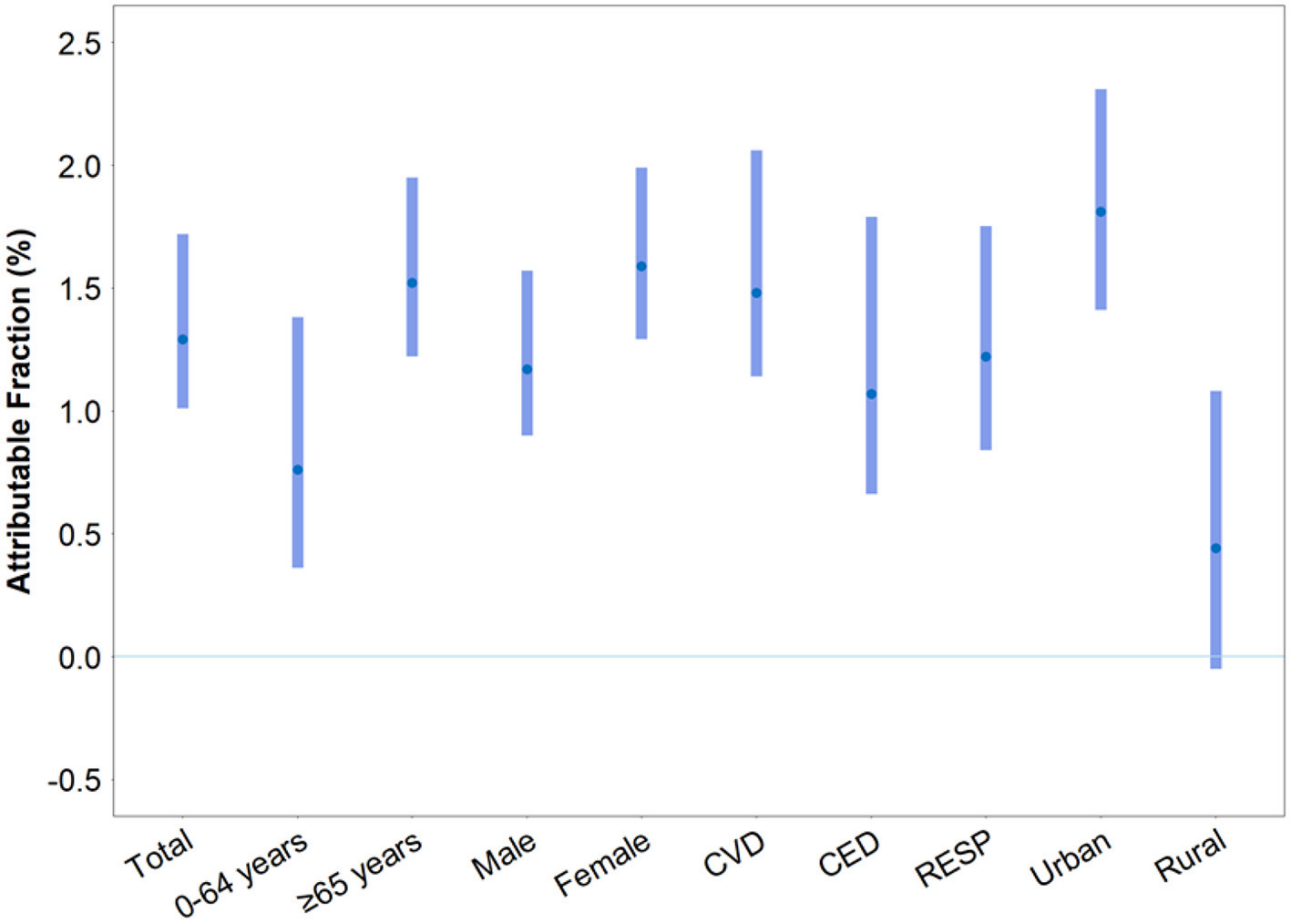
Attributable fraction (AF) of mortality caused by the Spring Festival period in China.

Figure 3 displayed the AF of mortality caused by the different phases of the Spring Festival. In total, 1.29% (95%CI: 1.01%, 1.72%) mortality was attributed to the Spring Festival effect. AFs were higher for people aged ≥ 65 years (AF: 1.52% [1.22%, 1.95%]) than for people aged <65 years (AF: 0.76% [0.36%, 1.38%]). AFs were also greater for women (AF: 1.59% [1.29%, 1.99%]) than for men (AF: 1.17% [0.90%, 1.57%]). For different diseases, AFs were 1.48% (1.14%, 2.06%) for people with CVD, 1.07% (0.66%, 1.79%) for people with CED, 1.22% (0.84%, 1.75%) for people with RESP. Furthermore, a greater AF was observed in people living in urban areas (AF: 1.81% [1.41%, 2.31%]) than in rural areas (AF: 0.44% [−0.05%, 1.08%]). For the different phases of the Spring Festival, we observed a similar pattern for AFs (Supplementary Table S1). Based on AFs and Chinese population data, we estimated that the Spring Festival could cause 15,410 excess deaths in China per year.

In addition, during this period of study, a U-shaped relationship between ambient temperature and mortality risk was observed (Supplementary Figure S2). However, we did not observe a significant association between PM_10_ and the mortality risk during the Spring Festival (ER: 0.08% [−0.01%, 0.17%]).

In the sensitivity analysis, we found that the results were relatively stable by changing degrees of freedom of time trend, a link function in DLNM, lag days of temperature and the Spring Festival, and the duration of the Spring Festival period (Supplementary Table S2).

## Discussion

In the current study, we observed that the Spring Festival was significantly related to elevated risk of mortality after controlling meteorological factors and air pollution. Interestingly, the mortality effect varied across the different phases that pre-Spring, mid-Spring, and post-Spring Festivals were significantly associated with a lower, an unchanged, and a higher mortality risk, respectively. We observed evidence of dip/rise death patterns during the Spring Festival in mainland China. In addition, we also found that the elderly aged over 64 years old, women, people with CVD, and people living in urban areas were more vulnerable to the Spring Festival.

Consistent with our findings, Phillips et al. found that Christmas/New Year's holidays in winter were the risk factors for cardiac and noncardiac mortality in the USA (5). Similarly, Knight found that cardiac mortality elevated during the Christmas holiday in summer in New Zealand (6). However, most of these studies focused on western festivals. Phillips and Smith have tried to explore the effect of the mid-Autumn Festival among Chinese in the USA. Unlike our findings, they just observed a postponement of death rather than a significantly increased mortality (3). The inconsistent findings may be explained by the following reasons: (1) the mid-Autumn Festival (3-day public holiday) is far inferior to the Spring Festival (7-day public holiday) in Chinese tradition; (2) Phillips's study was conducted among Chinese in the USA rather than Chinese in mainland China; (3) the mid-Autumn Festival is not an official holiday in the USA; and (4) some important confounders like temperature, humidity, and air pollution were not controlled in Phillips's study.

Given that the current study integrated mortality data, meteorological data, and air pollution from 285 locations across mainland China, the mortality risk of the Spring Festival was explored after controlling temperature, humidity, and air pollution. Our findings observed that pre-Spring, mid-Spring, and post-Spring Festivals were significantly associated with a lower, an unchanged, and a higher mortality risk, respectively. In other words, there was a dip/rise death pattern during the Spring Festival, which was consistent with previous studies (3, 5, 6). The reduced mortality in pre-Spring Festival may be attributed to the cultural and psychological factors. In Chinese culture, it is believed that a crucial occasion, such as a traditional festival or a person's birthday, may motivate a dying person to live beyond that day. A previous study also indicated that a person's psychological outlook of life can prolong or shorten life (15). In addition, we also found that the elevated mortality risk in the Spring Festival mainly occurred in the post-Spring Festival period. This finding can be explained by a triple-strike mechanism. People spend a lot of energy in traditional preparation before the festival after which the revelry during the holiday gives another blow to the unrecovered body. The traditional culture and limited medical resource additionally delay the timing of treatment during the festival. Although the body stress and compensatory function may keep the body working in the pre- and mid-Spring Festival period, a cumulative strike could consequently cause decompensation and death in post-Spring Festival.

To investigate whether increased deaths during post-Spring Festival were due to mortality postponement, we further estimated the total effect of the Spring Festival and found a significant effect of the Spring Festival on mortality. We further estimated that 15,410 excess deaths could be attributable to the Spring Festival in China per year. These findings suggested that the Spring Festival in mainland China may be a risk of mortality, which deserved further studies for the policy making of effective intervention.

Previous studies on the western festival have proposed a series of possible explanations such as: cold temperature, emotional stress, changes in life style, increased air pollution, postponement of death, and a delay in seeking medical care. Based on the multisource data and advanced statistical model, our study controlled some potential confounders and highlighted three possible mechanisms: (1) changing of life style: during the Spring Festival, people would break their daily routines and do some unhealthy behaviors, such as excessive drinking and overeating, irregular rest, and substance abuse (caffeine and methamphetamine) (16, 17). Epidemiology studies also indicated that the onset of acute pancreatitis (commonly due to overeating and overdrinking) peaked and blood pressure increased during the Spring Festival (18, 19). (2) Increased emotional stress: people could feel stress from having to interact with relatives, having to absorb financial pressures (expenses on gifts, entertaining, decorating, etc.) (20). The study of brain science further provided evidence that there was a “Christmas spirit network” in the human brain comprising several cortical areas (21). The Spring Festival-related emotional change could increase the risks of morbidity and mortality especially for cardio-cerebral vascular accidents (22). (3) Delayed treatment: Old Chinese people were not willing to go to hospital in the Spring Festival because they thought it would reduce their fortunes in the New Year. Moreover, insufficient medical workers during the Spring Festival vacation may also lead to delayed treatment. Previous studies have shown that patients are more likely to die in the hospital on the holiday/weekend than normal days (23, 24).

The present study also found that the elderly aged over 64 years old and women have a higher mortality risk during the Spring Festival. One of the probable reasons is that the elderly and women undertake most of housework during the most important festival in China. According to the Chinese National Time Use Survey (25), people aged 65–74 spend a longer time in housework (2.17 h per day) than other age groups, and women do more housework than men (2.10 vs. 0.75 h per day). Another reason for the vulnerability of old Chinese is that their aging body is vulnerable and they commonly refuse to go for treatment in the Spring Festival. Moreover, we also observed that people with CVD were at a higher risk of dying during the Spring Festival, which was consistent with previous studies (5, 6, 26). This could also be explained by the changes in diet and alcohol consumption and an inappropriate delay in seeking medical care. Further, we found a greater mortality effect of the Spring Festival in urban areas than rural areas. One of the possible explanations could be a higher prevalence of chronic disease of the elderly in urban areas than rural areas (for hypertension: 59.8 vs. 57.1%; for diabetes: 23.7 vs. 16.0%; for dyslipidemia: 44.2 vs. 31.8%, for myocardial infarction: 2.5 vs. 1.8%; for stroke: 5.7 vs. 4.1%; and for cancer: 2.9 vs. 2.1%) (27). The prevalence of the elderly with at least two chronic diseases was also greater in urban areas (40.2 vs. 31.3%) (27). According to our findings, young people and men should shoulder more housework for their elder and wife. In addition, old people with chronic disease, especially those living in urban areas, should avoid excessive drinking and eating during the Spring Festival.

The current study has several major strengths. First, the current study used the largest database in mainland China to explore the mortality risk of the Spring Festival. Second, multi-source data enabled us to control confounders like temperature, humidity, air pollution, etc., and provided robust evidence on the adverse effect of the Spring Festival. While this study has several strengths, some limitations should also be acknowledged. First, this study was an ecological study in essence and some biases were inevitable. Second, the current study did not include the study locations in western China. Third, we just used PM_10_ as an indicator of air pollution and its control in the model, which may not fully control the confounding effects of air pollution. Considering the close correlation between PM_10_ and other air pollutants (28), this is a feasible method to control air pollution and widely used in previous studies (9, 11, 29).

## Conclusion

In conclusion, the present study found that the Spring Festival increased the mortality risk and burden with high vulnerability in the elderly, women, people with CVD, and people living in urban areas. These findings will be informative in raising the awareness of self-protection as well as the development of health policies to alleviate the mortality burden related to the Spring Festival.

## Data Availability Statement

The data that supports the findings of this study are available on reasonable request from the corresponding author WJM. The data is not publicly available because the datasets are the intellectual and labor property of all institutions involved in this study, which cannot be accessed by the public without the permission of all the involved institutions.

## Ethics Statement

This study was approved by the Ethics Committee of Guangdong Provincial Center for Disease Control and Prevention (2019025). Data were analyzed at aggregate level and no participants were contacted.

## Author Contributions

WJM, GHH, MC, KP, and LS contributed to the conceptualization. WJM, GHH, and MC performed the statistical analysis and took the lead in drafting the manuscript and interpreting the results. RLM, JXH, ZLH, CLZ, XJX, YZX, MY, BH, LFL, TL, JPX, WWG, RYH, JHL, DHJ, MFQ, QLZ, YQX, WLZ, XL, and CRH provided the data and contributed to the interpretation. WJM and GHH revised the manuscript with advice from all authors. XFY took the lead in statistical analysis and drafted the revised manuscript during the review process. All authors contributed to the development of the manuscript and approved the final draft.

## Funding

This work was funded by the National Key Research and Development Program of China (2018YFA0606200), National Natural Science Foundation of China (42075173), Natural Science Foundation of Guangdong, China (2016A030313216, 2019A1515011880), Guangzhou Science and Technology Project (201704020194), and the Guangdong Health Innovation Platform, National Health and Medical Research Council Early Career Fellowship (GNT1139826).

## Conflict of Interest

The authors declare that the research was conducted in the absence of any commercial or financial relationships that could be construed as a potential conflict of interest.

## Publisher's Note

All claims expressed in this article are solely those of the authors and do not necessarily represent those of their affiliated organizations, or those of the publisher, the editors and the reviewers. Any product that may be evaluated in this article, or claim that may be made by its manufacturer, is not guaranteed or endorsed by the publisher.
