# Supplementary material for: The Spring Festival Is Associated With Increased Mortality Risk in China: A Study Based on 285 Chinese Locations
Source: Front Med (Lausanne). 2022 Mar 2;9:761060. doi: 10.3389/fmed.2022.761060 (PMC8924482; doi:10.3389/fmed.2022.761060)
Supplement: Supplementary file 1 [file Data_Sheet_1.docx]

**Supplement Materials**


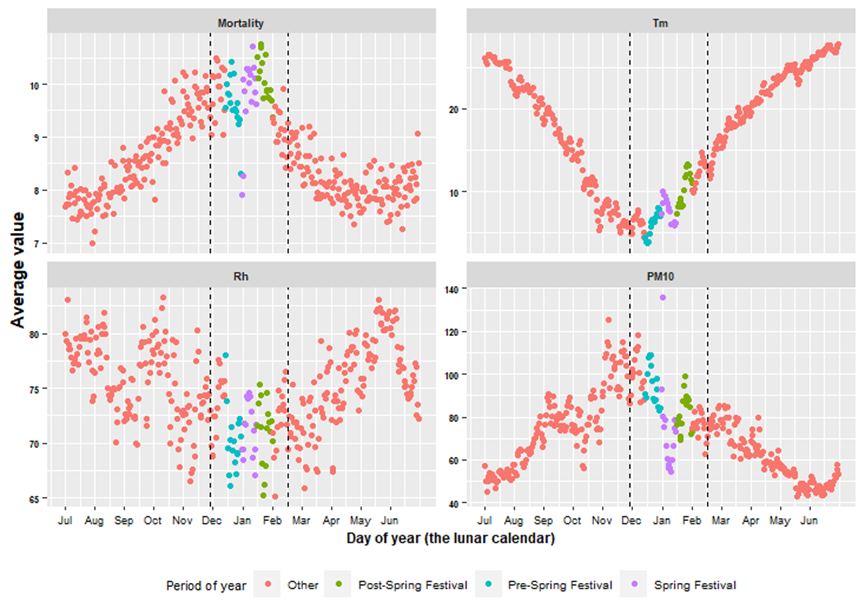


Figure S1 Daily average value distribution during 2013-2017 of mortality, temperature, relative humidity and PM_10_


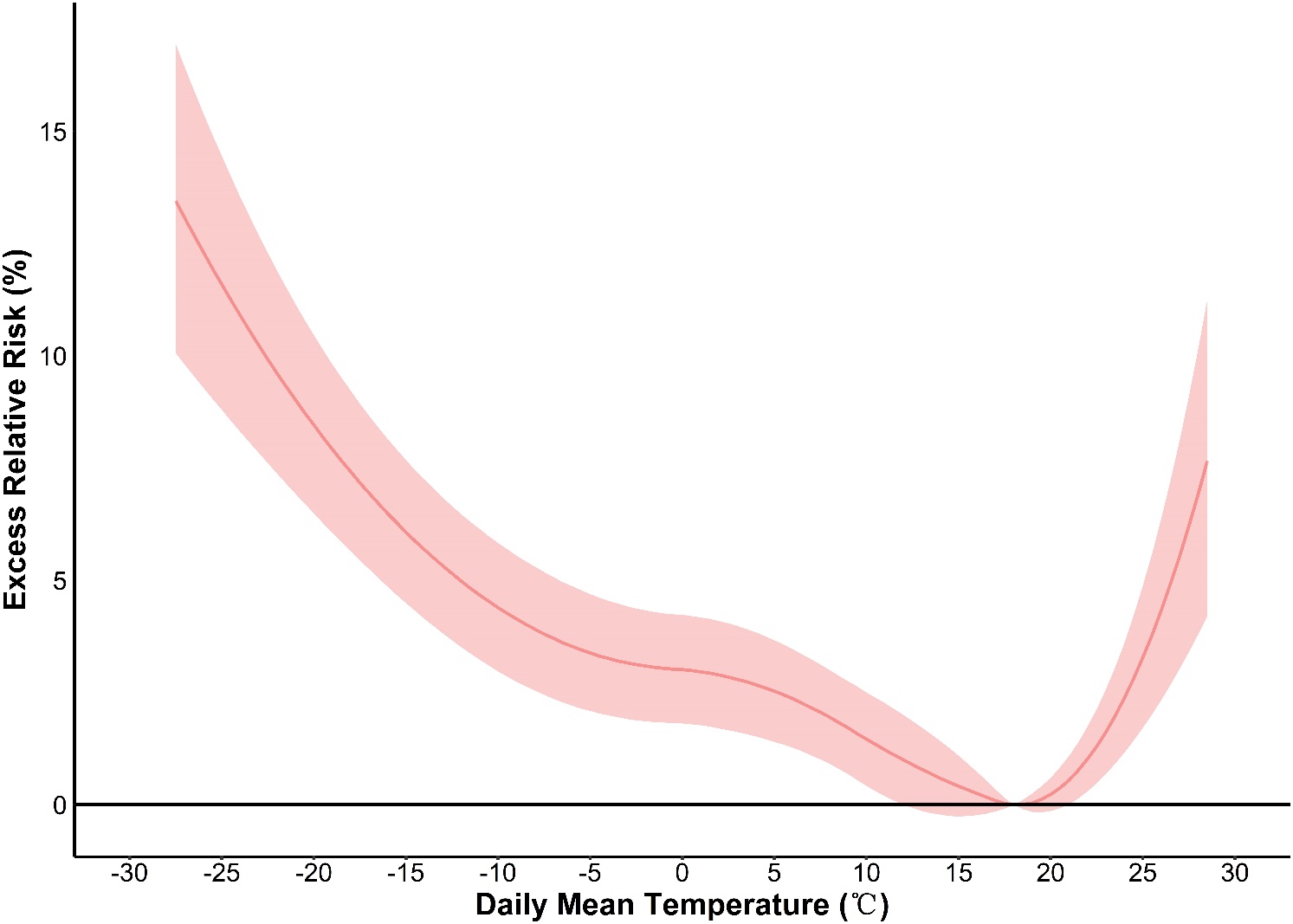


Figure S2 Exposure-Response Relationships between Daily Mean Temperature and Deaths during Spring Festival period.

Table S1 Attributable Fraction(AF) of mortality caused by different parts of the Spring Festival period in China

|  | **Pre-Spring Festival** | **Spring Festival** | **Post-Spring Festival** | **Total** |
| --- | --- | --- | --- | --- |
| **Total** | -1.71(-2.16,-1.07)^*^ | -0.40(-0.80,0.13) | 4.07(3.67,4.67)^*^ | 1.29(1.01,1.72)^*^ |
| **Age(year)** |  |  |  |  |
| 0- | -2.57(-3.23,-1.35)^*^ | -1.21(-1.70,-0.41)^*^ | 3.21(2.59,4.53)^*^ | 0.76(0.36,1.38)^*^ |
| 65- | -1.49(-2.00,-0.65)^*^ | -0.31(-0.84,0.45) | 4.33(3.84,5.09)^*^ | 1.52(1.22,1.95)^*^ |
| **Gender** |  |  |  |  |
| Male | -2.04(-2.50,-1.43)^*^ | -1.44(-1.59,-1.28)^*^ | 3.37(2.94,3.91)^*^ | 1.17(0.90,1.57)^*^ |
| Female | -1.39(-1.89,-0.66)^*^ | 0.53(0.10,1.05)^*^ | 5.03(4.60,5.65)^*^ | 1.59(1.29,1.99)^*^ |
| **Death of Cause** |  |  |  |  |
| CVD | -2.06(-2.58,-1.11)^*^ | -0.21(-0.86,0.95) | 4.86(4.34,5.78)^*^ | 1.48(1.14,2.06)^*^ |
| CED | -1.53(-2.14,-0.20)^*^ | -0.07(-0.41,0.41) | 3.62(3.2,4.21)^*^ | 1.07(0.66,1.79)^*^ |
| RESP | -0.22(-0.89,1.14) | -1.28(-1.74,-0.65)^*^ | 1.92(1.5,2.49)^*^ | 1.22(0.84,1.75)^*^ |
| **Region** |  |  |  |  |
| Urban | 0.97(0.47,1.64) | -0.85(-1.43,-0.14)^*^ | 2.54(1.93,3.31)^*^ | 1.81(1.41,2.31)^*^ |
| Rural | -5.96(-6.78,-4.75)^*^ | 0.25(-0.47,1.11) | 6.21(5.34,7.33)^*^ | 0.44(-0.05,1.08) |

* P < 0.05

Table s2 Sensitivity analysis of mortality risk associated with different parts of the Spring Festival period by changing degree of freedom of time trend, lag days of temperature or Spring Festival, and duration of the Spring Festival period

|  | **Pre-Spring Festival** | **Spring Festival** | **Post- Spring Festival** | **Total** |
| --- | --- | --- | --- | --- |
| **df for time trend** |  |  |  |  |
| 1 | -0.24(-1.57,1.11) | 2.93(1.93,3.94)^*^ | 5.39(4.24,6.56)^*^ | 3.07(2.35,3.8)^*^ |
| 2 | -1.58(-3.09,-0.05)^*^ | -0.4(-2.01,1.23) | 3.63(2.15,5.12)^*^ | 2.11(0.91,3.33)^*^ |
| 3 | -4.14(-5.5,-2.76)^*^ | -0.7(-2.29,0.92) | 5.52(4.10,6.95)^*^ | 1.27(0.01,2.55)^*^ |
| **Lag days of Spring Festival** | |  |  |  |
| 0 | -1.58(-3.09,-0.05)^*^ | -0.4(-2.01,1.23) | 3.63(2.15,5.12)^*^ | 2.11(0.91,3.33)^*^ |
| 1 | -1.03(-2.65,0.62) | 1.99(0.12,3.89)^*^ | 4.00(2.37,5.67)^*^ | 1.94(0.63,3.27)^*^ |
| **Lag days of temperature** | |  |  |  |
| 0 | -1.58(-3.09,-0.05)^*^ | -0.4(-2.01,1.23) | 3.63(2.15,5.12)^*^ | 2.11(0.91,3.33)^*^ |
| 1 | -1.48(-3.09,0.16) | 2.09(0.21,4.01)^*^ | 4.22(2.56,5.90)^*^ | 1.71(0.39,3.06)^*^ |
| **Duration of the Spring Festival period** | |  |  |  |
| 10^†^ | -0.73(-2.69,1.27) | 1.69(-0.62,4.05) | 4.07(2.33,5.84)^*^ | 2.02(0.61,3.45)^*^ |
| 15^&^ | -0.54(-2.19,1.14) | 5.84(4.10,7.61)^*^ | 7.21(5.64,8.79)^*^ | 2.47(1.20,3.75)^*^ |
| 16^#^ | -1.58(-3.09,-0.05)^*^ | -0.4(-2.01,1.23) | 3.63(2.15,5.12)^*^ | 2.11(0.91,3.33)^*^ |

† the Spring Festival period: from the New Year's Eve to the 9th day of the Lunar calendar.

$ the Spring Festival period: from the first day to the 15th day of the Lunar calendar

# the Spring Festival period: from the New Year's Eve to the 15th day of the Lunar calendar

* P < 0.05

Table S3 Excess relative risk (%) during different phases of the Spring Festival period in China compared with control period (Poisson link).

|  | **Excess Relative Risk (%) and 95% CI** | | | |
| --- | --- | --- | --- | --- |
|  | Pre-Spring Festival^a^ | Mid-Spring Festival^b^ | Post-Spring Festival^c^ | Total Spring Festival^d^ |
| **Total** | -1.92(-3.62,-0.19)^*^ | -0.46(-2.35,1.47) | 3.85(2.17,5.56)^*^ | 2.18(0.83,3.54)^*^ |
| **Age(year)** |  |  |  |  |
| 0- | -2.59(-4.94,-0.18)^*^ | -1.19(-4.03,1.73) | 3.11(0.64,5.65)^*^ | 1.41(-0.62,3.49) |
| 65- | -1.63(-3.49,0.26) | -0.20(-2.29,1.94) | 4.31(2.52,6.12)^*^ | 2.61(1.15,4.10)^*^ |
| **Gender** |  |  |  |  |
| Male | -2.00(-3.86,-0.11)^*^ | -1.30(-3.42,0.87) | 3.34(1.53,5.17)^*^ | 2.06(0.58,3.57)^*^ |
| Female | -1.69(-3.67,0.33) | 0.57(-1.87,3.07) | 4.87(2.84,6.94)^*^ | 2.67(1.06,4.31)^*^ |
| **Death of Cause** |  |  |  |  |
| CVD | -1.48(-3.64,0.72) | -0.01(-2.67,2.72) | 4.32(2.18,6.51)^*^ | 2.60(0.84,4.40)^*^ |
| CED | -1.91(-4.71,0.97) | -0.08(-3.39,3.34) | 3.87(1.36,6.44)^*^ | 1.77(-0.52,4.12) |
| RESP | -0.39(-3.32,2.63) | -1.43(-4.76,2.01) | 1.83(-0.69,4.42) | 2.07(-0.25,4.45) |
| **Region** |  |  |  |  |
| Urban | 0.29(-1.45,2.07) | -1.31(-3.52,0.94) | 2.72(0.82,4.64)^*^ | 3.23(1.66,4.82)^*^ |
| Rural | -4.79(-7.84,-1.65)^*^ | 0.63(-2.67,4.04) | 5.22(2.22,8.30)^*^ | 0.65(-1.68,3.02) |

^a^ Mid-Spring Festival: from Lunar New Year’s Eve to Lantern Festival (the 15th day of the first lunar month).

^b^ Pre-Spring Festival: 16 days before the mid-Spring Festival.

^c^ Post-Spring Festival: 16 days after the mid-Spring Festival.

^d^ Total Spring Festival: Mid-Spring Festival+ Pre-Spring Festival+ Post-Spring Festival.

* P < 0.05
